# Cysteine Boosts Fitness Under Hypoxia-Mimicked Conditions in Ovarian Cancer by Metabolic Reprogramming

**DOI:** 10.3389/fcell.2021.722412

**Published:** 2021-08-11

**Authors:** Sofia C. Nunes, Cristiano Ramos, Inês Santos, Cindy Mendes, Fernanda Silva, João B. Vicente, Sofia A. Pereira, Ana Félix, Luís G. Gonçalves, Jacinta Serpa

**Affiliations:** ^1^Centro de Estudos de Doenças Crónicas, NOVA Medical School/Faculdade de Ciências Médicas, Universidade Nova de Lisboa, Lisbon, Portugal; ^2^Instituto Português de Oncologia de Lisboa Francisco Gentil, Lisbon, Portugal; ^3^Instituto de Tecnologia Química e Biológica António Xavier, Universidade Nova de Lisboa, Oeiras, Portugal

**Keywords:** carbon source, cysteine, hypoxia, ovarian cancer, hydrogen sulfide, cystine, bioenergetics, microenvironment

## Abstract

Among gynecologic malignancies, ovarian cancer is the third most prevalent and the most common cause of death, especially due to diagnosis at an advanced stage together with resistance to therapy. As a solid tumor grows, cancer cells in the microenvironment are exposed to regions of hypoxia, a selective pressure prompting tumor progression and chemoresistance. We have previously shown that cysteine contributes to the adaptation to this hypoxic microenvironment, but the mechanisms by which cysteine protects ovarian cancer cells from hypoxia-induced death are still to be unveiled. Herein, we hypothesized that cysteine contribution relies on cellular metabolism reprogramming and energy production, being cysteine itself a metabolic source. Our results strongly supported a role of xCT symporter in energy production that requires cysteine metabolism instead of hydrogen sulfide (H_2_S) *per se*. Cysteine degradation depends on the action of the H_2_S-synthesizing enzymes cystathionine β-synthase (CBS), cystathionine γ-lyase (CSE), and/or 3-mercaptopyruvate sulfurtransferase (MpST; together with cysteine aminotransferase, CAT). In normoxia, CBS and CSE inhibition had a mild impact on cysteine-sustained ATP production, pointing out the relevance of CAT + MpST pathway. However, in hypoxia, the concomitant inhibition of CBS and CSE had a stronger impact on ATP synthesis, thus also supporting a role of their hydrogen sulfide and/or cysteine persulfide-synthesizing activity in this stressful condition. However, the relative contributions of each of these enzymes (CBS/CSE/MpST) on cysteine-derived ATP synthesis under hypoxia remains unclear, due to the lack of specific inhibitors. Strikingly, NMR analysis strongly supported a role of cysteine in the whole cellular metabolism rewiring under hypoxia. Additionally, the use of cysteine to supply biosynthesis and bioenergetics was reinforced, bringing cysteine to the plateau of a main carbon sources in cancer. Collectively, this work supports that sulfur and carbon metabolism reprogramming underlies the adaptation to hypoxic microenvironment promoted by cysteine in ovarian cancer.

## Introduction

Despite all the progresses developed in prevention and new treatment approaches, cancer corresponds to the second leading cause of death worldwide (Fitzmaurice et al., 2015). Ovarian cancer is not an exception to this scenario, being expected, in 2020, 0.28 million new cases and 0.18 million ovarian cancer deaths worldwide (Ferlay et al., 2013). The late diagnosis together with resistance to conventional therapy represent the major causes for the poor prognosis of this disease (Jayson et al., 2014).

Epithelial ovarian cancer (EOC) includes most (90%) ovarian malignancies (Bast et al., 2009; Desai et al., 2014) that can be classified based on histopathology and molecular/genetic features, being mainly classified as serous low-grade (LG-OSC, <5%) and high-grade (HG-OSC, 70%), endometrioid (OEC, 10%), clear cell (OCCC, 10%) and mucinous (OMC, 3%) (Prat, 2012; Reid et al., 2017).

As a solid tumor grows, cancer cells are exposed to varying oxygen tensions and to different degrees of hypoxia, being these oxygen fluctuations strongly linked to oxidative stress (Fruehauf and Meyskens, 2007; Saed et al., 2017) and known to be responsible for tumor progression and resistance to therapy (Vaupel and Mayer, 2007; Semenza, 2012). In ovarian cancer, oxidative stress was already associated with the pathogenesis of the disease (Senthil et al., 2004; Saed et al., 2017), indicating that ovarian cancer cells present mechanisms that allow them to cope with the harmful oxidative conditions. We disclosed that cysteine facilitates the adaptation of ovarian cancer cells to hypoxic environments and to carboplatin-induced death (Nunes et al., 2018a,b). Moreover, the relevance of cysteine in the clinical context of ovarian cancer was also corroborated, since ascitic fluid from ovarian cancer patients – an important compartment of tumor microenvironment – showed cysteine as the prevalent thiol and because cysteine levels were also altered in serum from patients with ovarian tumors (Nunes et al., 2018b). Cysteine role in cancer cells survival was already associated with its role as a precursor of the antioxidant glutathione (GSH) (Schnelldorfer et al., 2000; Balendiran et al., 2004; Lopes-Coelho et al., 2016) and due to hydrogen sulfide (H_2_S) generation (Bhattacharyya et al., 2013; Szabo et al., 2013; Pan et al., 2015; Panza et al., 2015; Sen et al., 2015; Gai et al., 2016; Szczesny et al., 2016) by cysteine catabolism through the activity of the enzymes cystathionine β-synthase (CBS), cystathionine γ-lyase (CSE), and/or 3-mercapto-pyruvate sulfurtransferase (MpST) together with cysteine aminotransferase (CAT) (Wang, 2012; Kabil and Banerjee, 2014; Giuffrè and Vicente, 2018; Hipólito et al., 2020). CBS, CSE, and MpST also catalyze the cyst(e)ine-dependent production of cysteine persulfide (CysSSH), which in several (patho)physiological contexts affords protection from damaging cysteine oxidation (e.g., Filipovic et al., 2018; Zivanovic et al., 2019; Zuhra et al., 2021). Another recently proposed pathway linking cysteine catabolism with mitochondrial bioenergetics concerns the mitochondrial isoform of cysteinyl-tRNA synthase (CARS2), which converts cysteine into CysSSH and also incorporates persulfidated cysteine into nascent polypeptides (Akaike et al., 2017; Bianco et al., 2019). The uptake of cysteine by cells seems to be preferentially mediated by xCT, a member of the cystine-glutamate transporter xc- system, known to mediate the uptake of cystine, the oxidized form of cysteine (Sato et al., 2000). Interestingly, it was reported that xCT was associated with intracellular GSH level and with cisplatin resistance in human ovarian cancer cell lines (Okuno et al., 2003). More recently, xCT has been implicated as part of highly favorable metabolic cancer phenotype, presenting increased capacity of ATP generation amongst other features pivotal for cancer cells survival and chemoresistance (Polewski et al., 2016; Jourdain et al., 2021).

Here, we aimed to address the mechanism by which cysteine protects ovarian cancer cells from hypoxia-induced death, hypothesizing that in addition to its role as GSH precursor, cysteine contributes to cell bioenergetics and biosynthesis under hypoxic conditions, as both a donor of H_2_S and as a carbon source. To test this hypothesis, we used two different ovarian cancer cell lines derived from two different histological types. ES2 cells correspond to an ovarian clear cell carcinoma (OCCC), an uncommon but highly chemoresistant histotype, and OVCAR3 cells correspond to a high-grade ovarian serous carcinoma (HG-OSC, the most frequent histotype), which usually acquires resistance during chemotherapy (Goff et al., 1996; Sugiyama et al., 2000; Cooke et al., 2011; Beaufort et al., 2014).

## Materials and Methods

### Cell Culture

Cell lines from OCCC (ES2; CRL-1978) and HG-OSC (OVCAR-3; HTB-161) were obtained from American Type Culture Collection (ATCC). Cells were maintained at 37°C in a humidified 5% CO_2_ atmosphere, and cultured in Dulbecco’s Modified Eagle medium (DMEM, 41965-039, Gibco, Life Technologies), containing 4.5 g/L of D-glucose and 0.58 g/L of L-glutamine, 1% FBS (S 0615, Merck), 1% antibiotic-antimycotic (P06-07300, PAN Biotech) and 0.1% gentamicin (15750-060, Gibco, Life Technologies). Cells were exposed either to 0.402 mM L-cysteine (102839, Merck) and/or to hypoxia induced with 0.1 mM cobalt chloride (CoCl_2_) (C8661, Sigma-Aldrich) as previously (Nunes et al., 2018a,b).

Prior to any experiment, cells were synchronized under starvation (culture medium without FBS) for 8 h at 37°C and 5% CO_2_.

### ATP Quantification

To test the effect of xCT inhibition on ATP levels, cells (5 × 10^5^ or 2.5 × 10^5^ cells/well) were seeded in 6-well white plates in hypoxia-mimic conditions induced with 0.1 mM CoCl_2_ and challenged with 0.25 mM sulfasalazine (S0883, Sigma), an xCT inhibitor (Gout et al., 2001). After 24 h, the medium was replaced and the challenging/stimulatory conditions reset. Cells were collected at 48 h after stimulation.

To test the effect of the enzymes involved in cysteine metabolism on ATP synthesis, cells (5 × 10^5^ or 2.5 × 10^5^ cells/well) were seeded in 6-well plates and cultured in control condition and exposed to 0.402 mM L-cysteine and/or 0.100 mM CoCl_2_. The previous conditions were combined with 1 mM *O*-(carboxymethyl)hydroxylamine hemihydrochloride (AOAA, C13408, Sigma) and 3 mM DL-propargylglycine (PAG, P7888, Sigma). AOAA is an inhibitor of both CBS and CSE, whereas PAG is selective toward CSE. After 24 h, the medium was replaced and the challenging/stimulatory conditions reset. Cells were collected at 16 and 48 h after stimulation. This assay was also performed with cell collection after 2 h of experimental conditions. In this case, 5 × 10^5^ cells were seeded in 6-well white plates for both ES2 and OVCAR3 cells.

To investigate the effect of cyst(e)ine and NaHS (H_2_S donor) on ATP synthesis upon xCT inhibition, cells (5 × 10^5^ or 2.5 × 10^5^ cells/well) were seeded in 6-well plates and exposed to hypoxia induced with 0.1 mM CoCl_2_ with and without 0.402 mM L-cysteine combined with 0.25 mM sulfasalazine, 30 μM NaHS (161527 Sigma-Aldrich) or both. After 24 h, the medium was replaced and the challenging/stimulatory conditions reset. Cells were collected at 48 h after stimulation. This assay was also performed with cell collection after 1 h of experimental conditions. In this case, 5 × 10^5^ cells were seeded in 6-well white plates for both ES2 and OVCAR3 cells.

Cells were scraped with cold PBS containing 2 mM EDTA and homogenized in 1% Non-idet P-40 (NP-40) lysis buffer (1% NP40 N-6507 Sigma, and 1× protease inhibitor, SIGMAFAST^TM^ Protease inhibitor cocktail tablets S8830, Sigma) on ice for 30 min and centrifuged at 20,000 × *g* for 5 min at 4°C. Protein was quantified and the same amount of total protein was used within each assay, in a range between 2.5 and 10 μg. ATP determination kit (A22066, Molecular probes) was used in accordance with the manufacturer’s instructions. The measurements were performed using the Luciferase protocol in a VIKTOR3 plate reader (PerkinElmer), using the Wallac 1420 software, version 3.0 (Luminometry, Luciferase FIR protocol). ATP concentration was determined against an ATP calibration curve, within the range between 0 and 30 μM.

### Immunofluorescence Analysis

Cells (1 × 10^5^ cells/well) were seeded in 24-well plates and cultured either in control or stimulated/challenged conditions. After 16 h of experimental conditions, cells were fixed with 4% paraformaldehyde for 15 min at 4°C and permeabilized with saponin 0.1% in PBS-BSA (PBS buffer containing 0.5%, w/v, bovine serum albumin) for 15 min at room temperature. Cells were then incubated with anti-xCT (ab175186, abcam) for 30 min (diluted 1:100 in PBS-BSA) and incubated with secondary antibody for 2 h at room temperature (Alexa Fluor^®^ 488 anti-rabbit, A-11034, Invitrogen) (1:1000 in PBS-BSA). The protocol was repeated for anti-TOMM20 (1:100 in PBS PBS-BSA; ab186734 from abcam) for 2 h at room temperature and incubated with secondary antibody for 2 h at room temperature (Alexa Fluor^®^ 594 anti-rabbit, A-11037, Invitrogen) (1:1000 in PBS-BSA).

The slides were mounted in VECTASHIELD media with 4′-6-diamidino-2-phenylindole (DAPI) (Vector Labs) and examined by standard fluorescence microscopy (Zeiss Imajer.Z1 AX10 microscope). Images were acquired and processed with CytoVision software.

### Western Blotting Analysis of Mitochondria Extracts

Cells (7 × 10^6^ cells/flask) were cultured in 150 cm^2^ culture flasks and submitted to control condition and to 0.402 mM L-cysteine and/or 0.1 mM CoCl_2_. Cells were collected after 16 h of experimental conditions, and mitochondria were isolated with the Mitochondria Isolation Kit for profiling cultured cells (MITOISO2, Sigma). Briefly, cells were scraped, centrifuged for 5 min at 600 × *g* and washed in ice cold PBS. The cell pellet was resuspended in 650 μL of Lysis Buffer, according to the manufacturer’s instructions.

The mitochondrial lysates were centrifuged at 20,817 × *g* for 5 min at 4°C and total protein concentration was determined based on the Bradford method (500-0006, Bio Rad). Western blotting for xCT and TOMM20 detection was performed with 15 μg of total protein, whereas for MpST, 20 μg of total protein were used. TOMM20 was used as a mitochondrial endogenous control.

Total protein in SDS-PAGE gels was transferred to PVDF membranes by semi-dry transfer. Membranes were blocked with PBS-milk (PBS containing 5% non-fat skimmed powdered milk) incubated with anti-xCT (1:1000 in PBS-milk; ab175186 from abcam) or anti-MpST (1:250 in PBS-milk; HPA001240 from Sigma) at 4°C with stirring, overnight. The membranes were then washed three times, for 5 min, with PBS 1 × 0.1% (v/v) Tween 20, and further incubated with secondary antibody (anti-rabbit 1:5,000 in PBS-milk; 31460 from Thermo Scientific) for 2 h at room temperature. For the membrane that was previously incubated with xCT, the protocol was repeated for anti-TOMM20 (1:1000 in PBS 1x-Milk 5%; ab186734 from abcam).

### Cell Death Analysis

To test whether the protective effect of cysteine under hypoxia was dependent on H_2_S production, cells (1 × 10^5^ cells/well) were seeded in 24-well plates. Cells were cultured in control condition and exposed to 0.402 mM L-cysteine and/or hypoxia induced with 0.1 mM CoCl_2_, combined to 1 mM *O*-(carboxymethyl)hydroxylamine hemihydrochloride (AOAA, C13408, Sigma), to 3 mM of DL-propargylglycine (PAG, P7888, Sigma), or the combination of both. Cells were collected 16 h after stimulation.

Cells were harvested by centrifugation at 153 × *g* for 3 min, cells were incubated with 0.5 μL FITC-Annexin V (640906, BioLegend) in 100 μL annexin V binding buffer 1 × 10 mM HEPES (pH 7.4), 0.14 M sodium chloride (NaCl), 2.5 mM calcium chloride (CaCl_2_) and incubated at room temperature and in the dark for 15 min. After incubation, samples were rinsed with 0.2% (w/v) BSA (A9647, Sigma) in PBS and centrifuged at 153 × *g* for 3 min. Cells were suspended in 200 μL of annexin V binding buffer 1× and 2.5 μL propidium iodide (50 μg/mL) were added. Acquisition was performed in a FACScalibur (Becton Dickinson). Data were analyzed with FlowJo software.

### Nuclear Magnetic Resonance (NMR) Analysis

To address the metabolic effects of cysteine under normoxia and hypoxia in ES2 and OVCAR3 cells, the levels of several metabolites were measured by ^1^H-NMR. Cells (6.5 × 10^6^ cells) were seeded in 175 cm^2^ culture flasks and cultured in control condition and exposed to 0.402 mM L-cysteine and/or hypoxia-mimicked conditions induced with 0.1 mM CoCl_2_. Cells and supernatants were collected at 48 h after stimulation and stored at −20°C.

To follow all the metabolites directly derived from cysteine, cells (6.5 × 10^6^ cells) were seeded in 175 cm^2^ culture flasks and cultured in control condition and exposed to 0.402 mM L-cysteine and/or hypoxia induced with 0.1 mM CoCl_2_. In this assay, a fully U-^13^C-labeled L-cysteine (CLM-1868-PK, Cambridge Isotopes Laboratories, Inc.) was used. Cells were collected at 48 h after stimulation and supernatants were collected at 12, 24, 36, and 48 h of incubation and stored at −20°C.

Cell extracts were performed with methanol and chloroform to separate organic and aqueous phases. After cold methanol mixture (4 mL methanol/1 g weight pellet), two volumes (vol) of water were added, mixed, and incubated for 5 min on ice. Chloroform (1 vol) was then added to the sample and mixed. Finally, 1 vol of water was added and samples were incubated for 10 min on ice, following centrifugation at 1,699 × *g* for 15 min at 4°C. Aqueous (upper) and organic (lower) phases were collected. After extraction of solvents by evaporation, using a Speed Vac Plus Scllon, the samples were suspended on KPi buffer (50 mM, pH 7.4) in deuterated water (D_2_O) with 4% (v/v) sodium azide (NaN_3_) and 0.097 mM of 3-(trimethylsilyl)propionic-2,2,3,3-d4 (TSP). Culture supernatants were also diluted in this solution at 1:10 ratio. The 1H-NMR (noesypr1d), 13C-NMR (zgdc) and 13C-1H-HSQC (hsqcetgpsisp2) was obtained at 25°C in an Avance 500 II+ (Bruker) spectrometer operating at 500.133 MHz, equipped with a 5 mm TCI(F)-z H-C/N Prodigy cryo-probe. The chemical shifts in aqueous sample were referred to the TSP. Topspin 4.0.7 (Bruker) was used for acquisition and spectra analysis. Compound identification was performed by resorting to the Human Metabolome database (HMDB)^1^ and Chenomx NMR suite software version 8.1 (Chenomx Inc.). Metabolites concentration was determined using Chenomx NMR suite software version 8.1 for 1H-NMR spectra (Chenomx Inc.). Quantification of the resonances in 13C-1H-HSQC spectra was done by calculate the 1D projections and integrate the resonances areas in Topspin 4.0.7, using the TSP resonance was reference.

### Statistical Analysis

All data are presented as the mean ± SD with the exception of ^1^H-NMR data, which are presented as the median with 25th to 75th percentiles. All the graphics were done using the PRISM software package (PRISM 6.0 for Mac OS X; GraphPad software, United States, 2013) and Microsoft Excel. Assays were performed with at least three biological replicates per treatment, with the exception of NMR assay with ^13^C-labeled L-cysteine for which only two biological replicates were performed for the majority of treatments, and hence no statistical analysis was conducted. For comparisons of two groups, a two-tailed independent-samples *t*-test was used. For comparison of more than two groups, one-way analysis of variance (ANOVA) with Tukey’s multiple-comparisons *post hoc* test was used. Statistical significance was established as *p* < 0.05. All statistical analyses were performed using the IBM Corp. Released 2013. IBM SPSS Statistics for Macintosh, Version 22.0. Armonk, NY: IBM Corp. software, with the exception of metabolic pathway analysis that was performed with MetaboAnalyst 4.0^2^.

## Results

### xCT Transporter Localizes in Ovarian Cancer Cells’ Mitochondria and Its Inhibition Impairs ATP Production Under Hypoxia, Which Is Further Rescued by Cysteine Supplementation

We have previously reported that cysteine supplementation was able to protect ovarian cancer cells from hypoxia-induced death (Nunes et al., 2018a,b). Herein, we hypothesized that an increase in intracellular cysteine facilitates this adaptation to hypoxia by an increase in ATP production.

**FIGURE 1:**
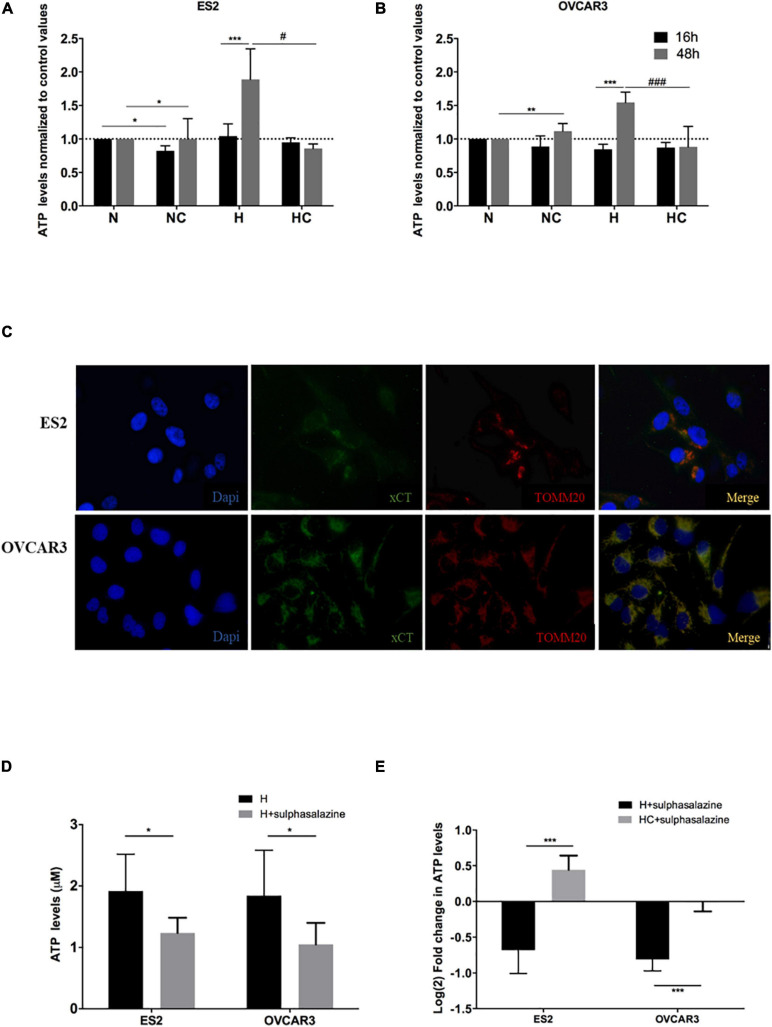
xCT transporter localizes in ovarian cancer cells’ mitochondria and its inhibition impairs ATP production under hypoxia, which is further rescued by cysteine. ATP levels normalized to control values (normoxia without cysteine supplementation) for 16 h and 48 h of experimental conditions for **(A)** ES2 cells and **(B)** OVCAR3 cells. The differences among treatments are pointed in the figure (one-way ANOVA with *post hoc* Tukey tests. For ES2, *n* = 6 for all treatments for 16 h of experimental conditions and *n* = 3 for 48 h of experimental conditions. For OVCAR3, *n* = 6 for all experimental conditions). **(C)** Immunofluorescence analysis for the xCT transporter and TOMM20 under control conditions (normoxia) for ES2 and OVCAR3 cells (*n* = 3). **(D)** ATP levels for 48 h of experimental conditions for ES2 and OVCAR3 cells under hypoxia and in the presence of the xCT inhibitor, sulfasalazine. **(E)** ATP levels for 48 h of experimental conditions for ES2 and OVCAR3 cells under hypoxia and in the presence of sulfasalazine and cysteine. The values were normalized to the respective control and log_2_ fold change was calculated. The differences among treatments are pointed in panels **(D,E)** (independent-samples *t*-test; *n* = 6 for all experimental conditions and for both cell lines). N, normoxia; NC, normoxia with cysteine; H, hypoxia; HC, hypoxia with cysteine. Results are shown as mean ± SD. *^/#^*p* < 0.05, **^/##^*p* < 0.01, ***^/###^*p* < 0.001.

Results show that after 16 h of experimental conditions, for ES2 cells, ATP levels did not differ among hypoxia with and without cysteine supplementation, and were higher under hypoxia without cysteine compared to normoxia with cysteine (*p* = 0.017) (Figure 1A). With 48 h of experimental conditions, ATP levels were increased under hypoxia without cysteine compared both to hypoxia (*p* = 0.018) and normoxia (*p* = 0.033) with cysteine (Figure 1A).

In OVCAR3 cells, after 16 h of experimental conditions, no differences were observed among treatments (Figure 1B). However, with 48 h of experimental conditions, the results were identical to ES2 cells, where cells cultured under hypoxia without cysteine presented higher ATP levels compared both to normoxia (*p* = 0.008) and hypoxia (*p* < 0.001) with cysteine (Figure 1B).

Therefore, after 48 h of experimental conditions, we observed increased ATP levels in hypoxia-mimicked conditions, which were prevented by cysteine administration in both ES2 and OVCAR3 (Figures 1A,B). These results support that ovarian cancer cells exhibit alternative ways that allow sustaining energy production under hypoxic environments. Since cells cultured under hypoxia without cysteine supplementation also present cysteine in the medium (yet in lower concentrations), and also because cells cultured under hypoxia with cysteine reach higher cellular confluence levels compared to hypoxia without cysteine, we cannot, nevertheless, rule out a role of cysteine in ATP production. Therefore, we further explored the role of xCT, a member of the cystine-glutamate transporter xc^–^ system, known to mediate the uptake of cystine (Sato et al., 2000) (the oxidized form of cysteine that is the predominant status of cysteine extracellularly), on ATP production under hypoxic environments. Thus, we started by addressing if this transporter could localize in ovarian cancer cells’ mitochondria thus suggesting a role of cysteine metabolism in ATP production via xc^–^ system. Immunofluorescence analysis suggested a mitochondrial localization of xCT in both ES2 and OVCAR3 cells (Figure 1C) that was further confirmed by western blotting analysis mitochondrial extracts (Supplementary Figure 1A). Interestingly, by western blotting with anti-TOMM20 (a mitochondrial marker) results show that ES2 cells present higher levels of TOMM20 under hypoxia compared to the other experimental conditions, whereas OVCAR3 cells seem to present the opposite tendency. As a consequence, the ratio xCT/TOMM20 was decreased under hypoxia-mimicked conditions in ES2 cells and increased in OVCAR3 cells (Supplementary Figure 1A).

We then explored the effect of xCT inhibition (with sulfasalazine) in the ability of ovarian cancer cells to produce ATP under hypoxia. Strikingly, xCT inhibition led to impaired ATP production in both cell lines (*p* = 0.038 for ES2 and *p* = 0.010 for OVCAR3) (Figure 1D). Importantly, cysteine was able to rescue this impaired ATP production upon xCT inhibition. Hence, we observed a significant increase in ATP levels upon cysteine supplementation (*p* < 0.001) (Figure 1E).

### CBS and CSE Inhibition Does Not Impair ATP Synthesis but Induces Cell Death in ES2 Cells

**FIGURE 2:**
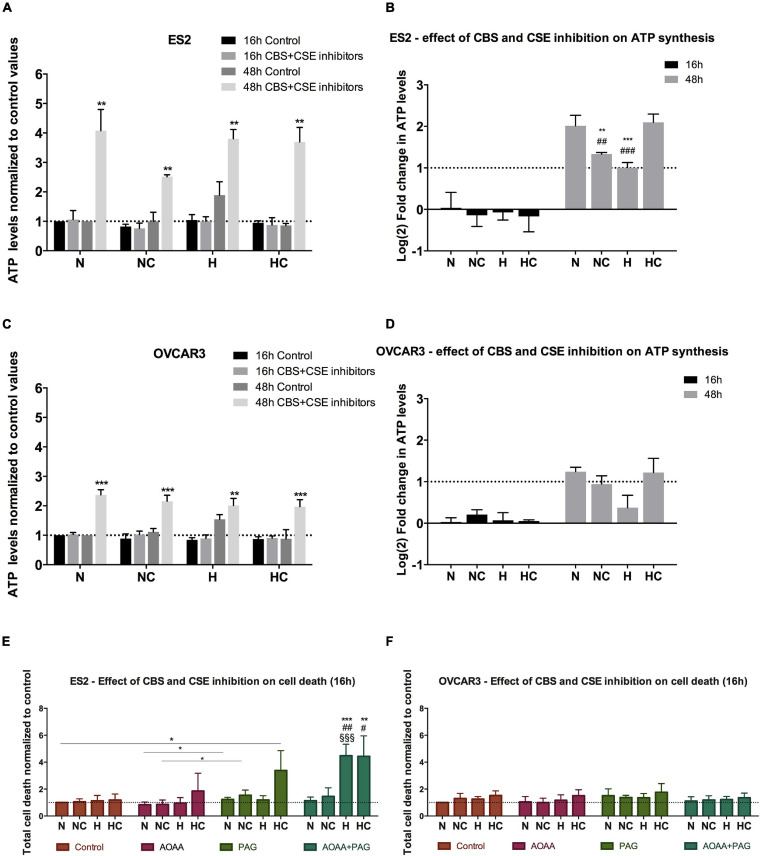
CBS and CSE inhibition does not impair ATP synthesis in ovarian cancer cells but affect ES2 cells survival, mainly in hypoxia-mimicked conditions. **(A–D)** ATP levels in the presence of 1 mM AOOA and 3 mM PAG for 16 h and 48 h of experimental conditions for ES2 [**A** – values normalized to control (normoxia without cysteine and without inhibitors) and **(B)** – values normalized to the respective control and log_2_ fold change calculation] and OVCAR3 [**C** – values normalized to control (normoxia without cysteine and without inhibitors) and **(D)** – values normalized to the respective control and log_2_ fold change calculation] cells. **(A,C)** The asterisks (*) represent the statistical significance compared to the respective control. **(B,D)** The asterisks (*) represent the statistical significance compared to N, the section symbols (§) represents the statistical significance compared to NC and the cardinals (#) represent statistical significance compared to HC. For ES2, *n* = 6 for all treatments for 16 h of experimental conditions and *n* = 3 for 48 h of experimental conditions. For OVCAR3, *n* = 6 for all experimental conditions. **(E,F)** Cell death levels in the control condition, in the presence of CBS/CSE inhibitor (1 mM AOAA), CSE inhibitor (3 mM PAG) or both inhibitors for ES2 **(E)** and OVCAR3 **(F)** cells. The asterisks (*) represent statistical significance between the respective treatments or between AOAA + PAG and its control, the cardinals (#) represent statistical significance between AOAA + PAG and AOAA alone and the section symbols (§) represent statistical significance between AOAA + PAG and PAG alone. For both cell lines, at least *n* = 6 were performed for all experimental conditions. N, normoxia; NC, normoxia with cysteine; H, hypoxia; HC, hypoxia with cysteine. Results are shown as mean ± SD. *p < 0.05, **p < 0.01, ***p < 0.001 [**(A,C)** independent-samples *t*-test; **(B,D–F)** one-way ANOVA with *post hoc* Tukey tests].

Since results suggested that cysteine has a role in ATP synthesis mediated by the xCT transporter, we next measured ATP levels upon CBS and CSE inhibition. Results have shown that after 16 h of experimental conditions, no differences were found in ATP levels with or without CSE (PAG) and CBS/CSE inhibitors (AOAA) for both ES2 and OVCAR3 cells in any culture condition (Figures 2A–D). As in Figure 1, prolonged (48 h) exposure to hypoxia of both ES2 and OVCAR3 cells resulted in increased ATP production. However, under the same conditions, cysteine decreased ATP levels in both cell lines. Given the dual nature of H_2_S as an electron supply for the mitochondrial ETC at low concentrations, or as an inhibitor of Complex IV at higher levels, this observation further suggests that exogenous cysteine affects ATP production via CBS/CSE-catalyzed H_2_S production.

Strikingly, upon exposure to CBS/CSE inhibitors for 48 h, increased ATP levels were observed in all experimental conditions for both ES2 (N *p* = 0.002, NC *p* = 0.001, H *p* = 0.004, HC *p* = 0.001) and OVCAR3 cells (N, NC and HC *p* < 0.0001 and H *p* = 0.003) with respect to their respective controls (48 h, no inhibitors; Figures 2A–D). In both cell lines exposed for 48 h to CBS/CSE inhibitors, hypoxia decreased significantly ATP production as compared to normoxia, but cysteine rescued this phenomenon (Figures 2B,D). These observations support the relevance of cysteine degradation on ATP production under hypoxia and put forward the possibility of a compensatory mechanism, e.g., via CAT/MpST or CARS2 to sustain H_2_S/CysSSH production under hypoxic conditions.

As ATP levels may increase under stressful conditions (e.g., Zhou et al., 2012) we investigate if there was an increase of cell death in the presence of CSE (PAG) and CBS + CSE (AOAA) inhibitors. We found that for ES2 cells, under normoxia both with and without cysteine supplementation (N and NC), CSE inhibition induced more cell death compared to joint CSE/CBS inhibition (*p* = 0.017) (Figure 2E). Under hypoxia (H), the combination of both inhibitors induced more cell death compared to their separate administration (*p* < 0.001). Interestingly, under hypoxia with cysteine, PAG alone resulted in increased cell death (*p* = 0.038 compared to the control) as well as in combination with AOAA (*p* = 0.002 compared to the control and *p* = 0.01 compared to AOAA alone) (Figure 2E). Regarding OVCAR3 cells, no differences were observed among treatments, with or without the inhibitors and/or cysteine (Figure 2F).

Together, results indicate that CBS and CSE inhibition, at least at the used doses, is not sufficient to impair ATP production but the inhibition of both enzymes affected ATP synthesis mainly under hypoxia for both cell lines. The role of MpST could not be addressed as no specific inhibitor is available.

Moreover, ES2 cells showed reduced viability upon the inhibition of both CBS and CSE under hypoxia-mimicked conditions, showing that ES2 cells are especially sensitive to the inhibition of these enzymes in this experimental condition, highlighting their role in hypoxia adaptation and the contribution of cysteine non-oxidative catabolism for cell survival in conditions of hypoxic stress.

### Cysteine Degradation Is Necessary to Rescue ATP Production Triggered by xCT Inhibition

Since results have suggested that cysteine has a role in ATP production under hypoxia-mimicked conditions that can be in part related to H_2_S synthesis, we investigated if the impact of cysteine on ATP production rely on CBS/CSE-catalyzed H_2_S production. For that we ascertained if cysteine metabolism was necessary or if a bolus addition of an H_2_S donor, as NaHS, would be sufficient to counteract the ATP impairment triggered by xCT inhibition, both at short (1 h) and long (48 h) exposure times.

Due to the unstable nature of NaHS that was reported in culture medium (Sun et al., 2017) leading to an instant release of H_2_S that decays rapidly (Hu et al., 2011), we evaluated ATP levels upon NaHS exposure for 1 h in the experimental conditions tested before. Fu et al. (2012) have reported that in vascular smooth-muscle cells, 1 h of NaHS exposure was sufficient to alter mitochondrial ATP production under hypoxia in a concentration-dependent manner, consistently with the dual stimulatory/inhibitory nature of H_2_S for mitochondrial bioenergetics.

**FIGURE 3:**
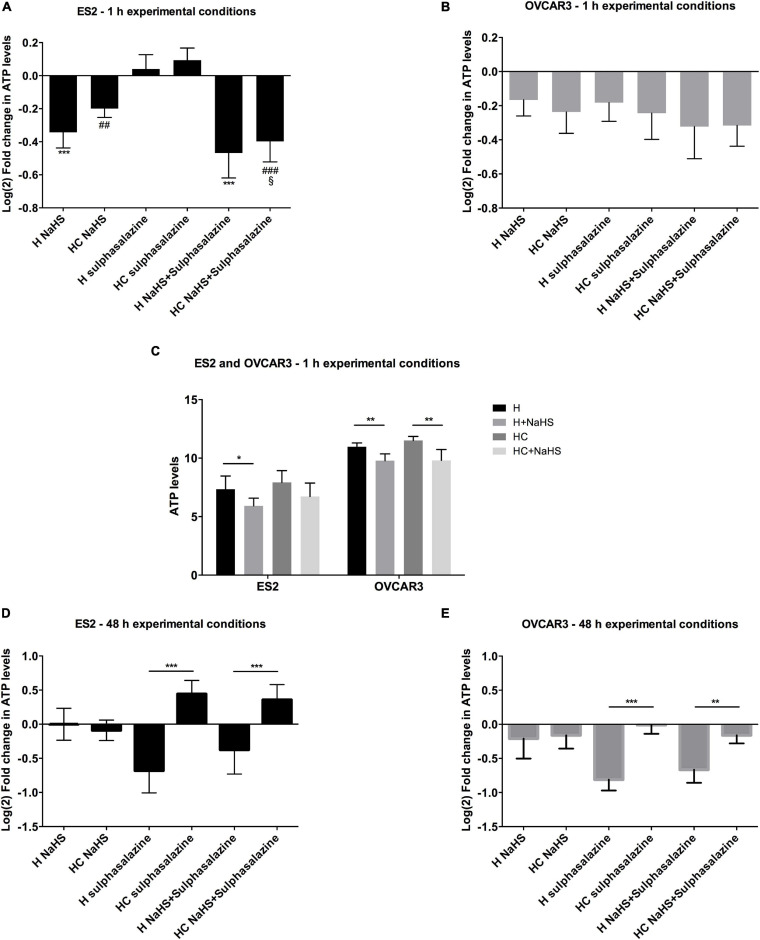
Cysteine, but not NaHS, is able to rescue the ATP synthesis impairment, after 48 h under hypoxia triggered by xCT inhibition. **(A–C)** ATP levels for 1 h of experimental conditions for **(C)**. ES2 and OVCAR3 cells under hypoxia with and without cysteine and in the presence of the H2S donor, NaHS sulfasalazine in which *n* = 5 for ES2; and H sulfasalazine and H NaHS C sulfasalazine, in which *n* = 5 for OVCAR3. **(D,E)** ATP levels for 48 h of experimental (un-normalized data), and **(D)** ES2 and **(E)** OVCAR3 cells under hypoxia with and without cysteine supplementation and in the presence of the xCT inhibitor, conditions for **(A)** ES2 and **(B)** OVCAR3 cells under hypoxia with and without cysteine and in the presence of the xCT inhibitor, sulfasalazine and the H2S donor, NaHS. Data were normalized to the respective control condition (the same environmental condition H/HC without NaHS or sulfasalazine). Data are presented as log2 fold change (*n* = 6 for both cell lines). H, hypoxia; HC, hypoxia with cysteine. Results are shown as mean ± SD. ^*/^*p* < 0.05, ^**/##^*p* < 0.01, and ^***/###^*p* < 0.001 [one-way ANOVA with *post hoc* Tukey tests for **(A,B,D,E)** and independent-samples *t*-test for **(C)**].

In hypoxic ES2 cells, within 1 h of conditions, while sulfasalazine alone tended to promote a slight increase in ATP (Figure 3A), 30 μM NaHS alone led to decreased ATP levels (*p* = 0.047) (Figure 3C), even upon xCT inhibition (H NaHS vs. H sulfasalazine *p* < 0.001; H NaHS + sulfasalazine vs. H sulfasalazine *p* < 0.001; HC NaHS vs. HC sulfasalazine *p* = 0.002; HC NaHS + sulfasalazine vs. HC sulfasalazine *p* < 0.001) (Figure 3A). In addition, under hypoxia with cysteine supplementation, NaHS did not affect ATP production (Figure 3C). For OVCAR3 cells, NaHS led to decreased ATP levels both under hypoxia with (*p* = 0.002) and without cysteine (*p* = 0.003) (Figure 3C), but no further differences were observed among treatments (Figure 3B). Furthermore, while 1 h of sulfasalazine was not sufficient to inhibit ATP levels in ES2 cells, there was a tendency for lower ATP levels in OVCAR3 cells (Figures 3A,B), suggesting that ES2 present higher basal levels of the xCT transporter or that these cells activate xCT (or other cysteine transporter) transcription in a more efficient way compared to OVCAR3 cells. In fact, in basal conditions, ES2 cells express higher xCT levels compared to OVCAR3 cells (Supplementary Figure 1B), hence supporting that a more prolonged exposure to sulfasalazine is necessary for the effective blocking of xCT in ES2 cells.

While the 1 h of experimental conditions is suitable to study NaSH effects, may not allow enough cysteine metabolism to permit relevant H_2_S production. In fact, with 48 h of experimental conditions, results have shown that whereas cysteine was able to revert the ATP impairment upon xCT inhibition in both ES2 and OVCAR3 cells (*p* < 0.001), NaHS alone (H_2_S donor) was not, as no differences were found with sulfasalazine with or without NaHS, for both cell lines (*p* > 0.05). These results might indicate that H_2_S by itself does not rescue ATP production, being cysteine degradation a requirement (Figures 3D,E).

### Cysteine Rescues Cellular Metabolism of Hypoxic ES2 Cells

**FIGURE 4:**
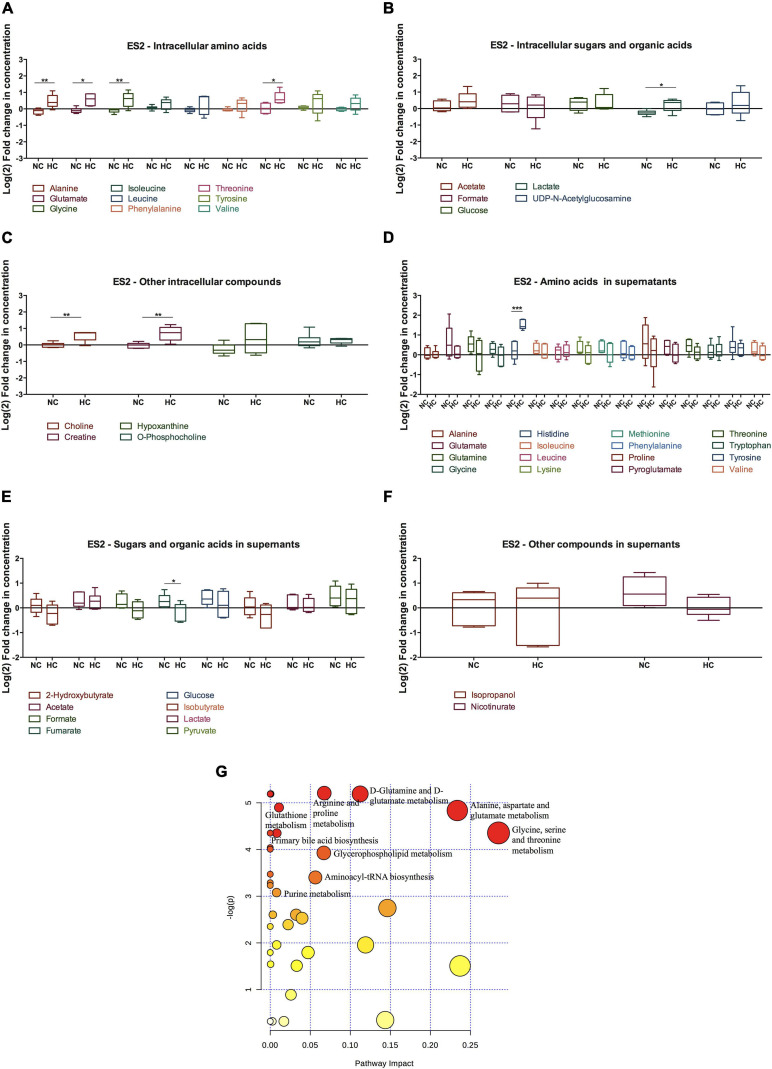
Cysteine rescues ES2 cellular metabolism and impacts several metabolic pathways under hypoxia. **(A–F)** Metabolites levels for 48 h of experimental conditions for ES2 cells. **(A)** Intracellular amino acids; **(B)** intracellular sugars and organic acids; **(C)** other intracellular metabolites; **(D)** amino acids in supernatants; **(E)** sugars and organic acids in supernatants; **(F)** other metabolites in supernatants. Data were normalized to the respective control condition (the same environmental condition NC/N and HC/H). NC, normoxia with cysteine; HC, hypoxia with cysteine. Results are shown as median with 25th to 75th percentiles. *p < 0.05, **p < 0.01, ***p < 0.001 (independent-samples *t-*test: NC, *n* = 6; HC, *n* = 5). **(G)** Metabolic pathway analysis for the effect of cysteine under normoxia and hypoxia in intracellular ES2 metabolites. All the matched pathways are displayed as circles. The color and size of each circle are based on *p*-value and pathway impact value, respectively. The most impacted pathways having statistical significance (*p* < 0.05) are indicated. Source: https://www.metaboanalyst.ca/Metabo Analyst/faces/home.xhtml.

**FIGURE 5:**
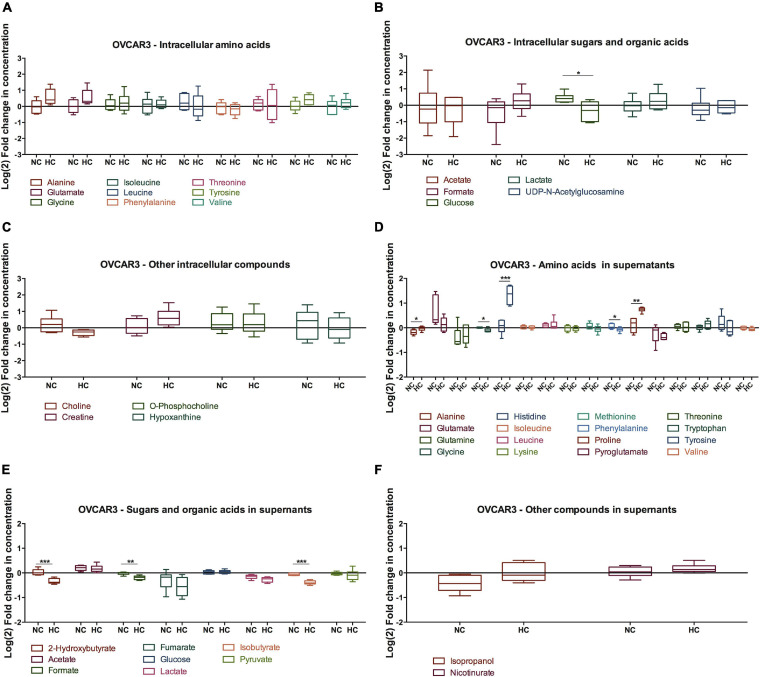
Effect of cysteine in OVCAR3 metabolites under normoxia and hypoxia. Metabolites levels for 48 h of experimental conditions for OVCAR3 cells. **(A)** Intracellular amino acids; **(B)** intracellular sugars and organic acids; **(C)** other intracellular metabolites; **(D)** amino acids in supernatants; **(E)** sugars and organic acids in supernatants; **(F)** other metabolites in supernatants. Data were normalized to the respective control condition (the same environmental condition NC/N and HC/H). NC, normoxia with cysteine; HC, hypoxia with cysteine. Results are shown as median with 25th to 75th percentiles. *p < 0.05, **p < 0.01, ***p < 0.001 (independent-samples *t*-test: NC, *n* = 6; HC, *n* = 6).

In order to address the metabolic effects of cysteine supplementation under normoxia and hypoxia in ES2 and OVCAR3 cells, we measured the levels of several metabolites by ^1^H-NMR (Figures 4, 5 and Supplementary Figure 2).

The intracellular metabolites were analyzed comparing the effect of cysteine in the metabolic profile of cells cultured in hypoxia with cells cultured in normoxia, in order to determine the variations in organic compounds driven by the presence of cysteine in both environments.

Regarding hypoxic ES2 cells, cysteine increased the intracellular levels of the amino acids alanine (*p* = 0.009), glutamate (*p* = 0.012), glycine (*p* = 0.008) and threonine (*p* = 0.016) (Figure 4A). Cysteine also led to increased intracellular levels of lactate (*p* = 0.021) (Figure 5B), choline (*p* = 0.005) and creatine (*p* = 0.005) (Figure 4C). In the extracellular media (supernatants), Co^2+^ employed to elicit hypoxia reacted as expected with histidine and therefore precluded any conclusion regarding histidine uptake (Supplementary Figure 2C). Cysteine decreased the release of fumarate under hypoxia (*p* = 0.046) (Figure 4E).

Through metabolic pathway analysis, results predicted that nine metabolic pathways are significantly and differently altered in ES2 cells by cysteine and hypoxia. The analysis showed alteration in the biosynthetic pathways of: (1) glycine, serine and/threonine; (2) alanine, aspartate and glutamate; (3) glutamine and glutamate; (4) arginine and proline; (5) GSH; (6) primary bile acid; (7) glycerophospholipid; (8) aminoacyl-tRNA; and (9) purine nitrogen bases (Figure 4G).

Regarding OVCAR3 cells, the only significant difference found on the effect of cysteine and hypoxia on intracellular metabolites concentration was on glucose levels, where there was a decrease in the intracellular levels of glucose (*p* = 0.021) (Figure 5). However, cysteine and hypoxia also provided a general tendency for increased intracellular amino acids such as alanine, glutamate, tyrosine and valine (Figure 5A). The measurement of the levels of these amino acids in the cell culture media showed that OVCAR3 cells uptake lower levels of alanine (*p* = 0.023) and proline (*p* = 0.008) and higher levels of glycine (*p* = 0.013) and phenylalanine (*p* = 0.049) due to the exposure to cysteine and hypoxia (Figure 5D). Cysteine and hypoxia also decreased the release of 2-hydroxybutyrate (*p* < 0.001), formate (*p* = 0.002) and isobutyrate (*p* < 0.001) (Figure 5E). As observed in ES2 cells, cysteine seemed to induce the release of glutamine, especially under normoxia (Supplementary Figure 2C).

The metabolite pathway analysis was not performed for OVCAR3 cells because cysteine and hypoxia only altered significantly one metabolite (glucose), thus making this analysis inaccurate.

Importantly, in the absence of cysteine, hypoxia did not alter the intracellular and extracellular levels of glucose and lactate in both cell lines, whereas it led to a decreased uptake of glutamine (*p* = 0.032) and to an increased release of fumarate (*p* = 0.041) in OVCAR3 cells (Supplementary Figure 2D).

### Cysteine Supplies Crucial Metabolic Pathways in ES2 Cells, Under Normoxia and Hypoxia

**FIGURE 6:**
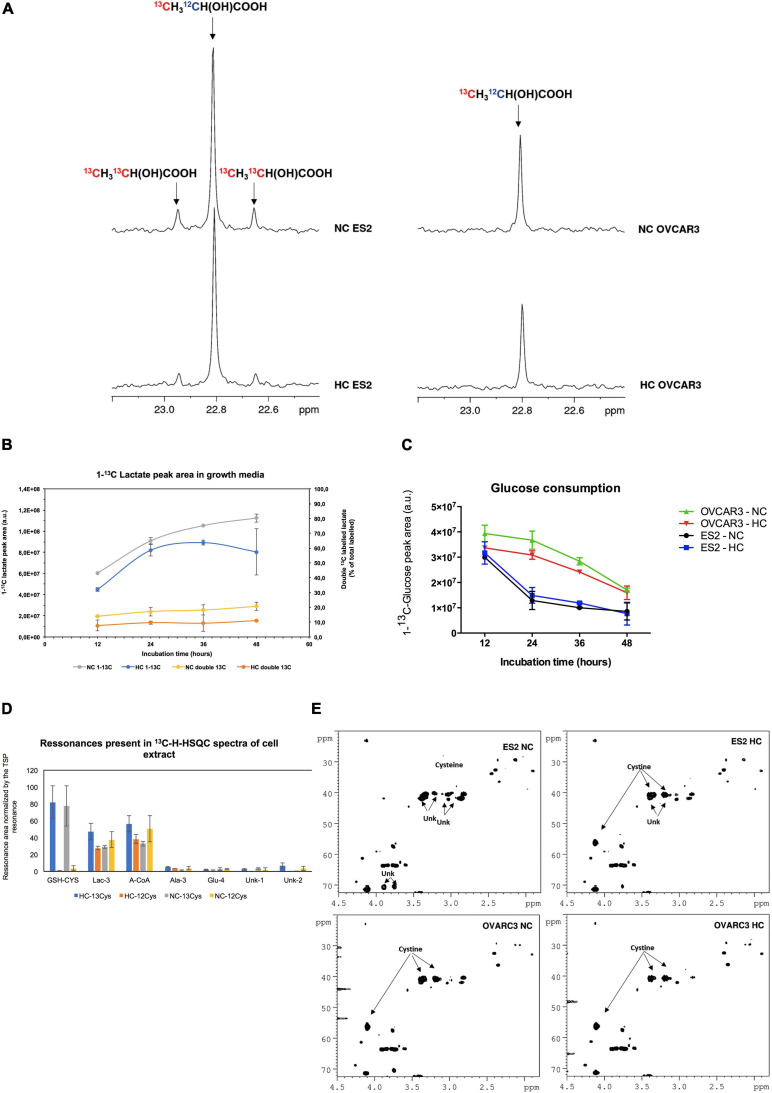
Cysteine is directly used by ES2 cells in the main metabolic pathways. **(A)** Highlight of the lactate methyl group resonance on the ^13^C-NMR spectra of 48 h culture media incubated with U-^13^C-cysteine. On the left panel, ES2 cells under normoxia (upper spectra) and under hypoxia (lower spectra); on the right panel, OVCAR3 cells under normoxia (upper spectra) and under hypoxia (lower spectra). **(B)** Production of lactate by ES2 cells in culture media at different time points (12, 24, 36, and 48 h). **(C)** Glucose consumption in ES2 and OVCAR3 cells. **(D)** Resonances areas of the ^13^C-1H-HSQC projection on ^13^C spectra normalized by TSP area in the cell extracts. **(E)** Highlight of ^13^C-1H-HSQC spectra of the growth media at 48 h incubation in the presence of U-^13^C-cysteine under normoxia and hypoxia conditions. NC, normoxia with cysteine; HC, hypoxia with cysteine. **(B–D)** Results are shown as mean ± SD. Assays were performed in biological triplicates.

To clarify the role of cysteine as a carbon source, we followed the metabolites directly derived from cysteine, by using ^13^C-L-cysteine. Our results supported that ES2 cells convert cysteine into lactate (Figures 6A,B). Whereas we observed the presence of satellite resonances indicating double ^13^C-labeled lactate (^13^CH_3_^13^CH(OH)COOH) in ES2 spectra, these were not observed in OVCAR3 (Figure 6A). The presence of ^13^C satellite resonances in the lactate is a direct result of ^13^C-L-cysteine conversion into lactate. Furthermore, total (single and double-labeled) ^13^C-lactate and the percentage of double-labeled ^13^C-lactate tended to be higher under normoxia (NC ^13^C and NC double ^13^C, respectively) compared to hypoxia (HC ^13^C and HC double ^13^C, respectively) (Figure 6B). We also observed that ES2 cells tended to consume glucose faster than OVCAR3 cells both under normoxia and hypoxia in the presence of cysteine (Figure 6C).

Furthermore, our results indicated that ES2 cells convert cysteine mainly in GSH (GSH-CYS), lactate (Lac-3) and acetyl-CoA (A-CoA). Small amounts of alanine (Ala3) and glutamate (Glu4) and of other two unidentified compounds (Unk1 and Unk2) were also detected (Figure 6D).

Finally, under normoxia, ES2 cells (ES2 NC) presented cysteine in solution instead of cystine, and produced different unknown compounds, while under hypoxia (ES2 HC) the main compound presented was cystine. In the case of OVCAR3 cells the profile was similar both under normoxia (OVCAR3 NC) and hypoxia (OVCAR3 HC), with cystine being the most representative compound (Figure 6E).

## Discussion

In this work, we provide novel data supporting that cysteine promotes sulfur and carbon metabolism reprogramming, underlying the adaptation to hypoxic microenvironment in ovarian cancer cells.

As a solid tumor grows, cancer cells are exposed to regions of hypoxia, long established as a stimulus for tumor progression and resistance to therapy (Vaupel and Mayer, 2007; Semenza, 2012). We have recently proposed that cysteine allows adaptation to hypoxic environments and also contributes to escape from carboplatin-induced death in ovarian cancer cells (Nunes et al., 2018a,b). In here, we aimed to investigate the mechanisms by which cysteine protects ovarian cancer cells under hypoxia, by addressing its role in cellular metabolism, namely through energy production.

Uptake of cystine, the oxidized form of cysteine, is mediated by xCT (solute carrier family 7 member 11 – SLC7A11), a member of the cystine-glutamate transporter xc^–^ system (Sato et al., 2000). Intracellularly, cystine is reduced to cysteine, which is the rate-limiting substrate for GSH synthesis, making xCT pivotal in the cellular redox balance maintenance (reviewed in Conrad and Sato, 2012). Herein, we have shown mitochondrial localization of the xCT transporter concomitant with impaired ATP production triggered by its pharmacological inhibition under hypoxia, thus indicating a role of the xc^–^ system via cystine uptake also in energy production. These data are in accordance with recent findings supporting a role of Nrf2 in the regulation of mitochondrial ATP synthesis (reviewed in Vomund et al., 2017). As a transcription factor, Nrf2 was already reported to regulate the expression of xCT and the activity of the xc^–^ system in response to oxidative stress in human breast cancer cells (Habib et al., 2015).

Cysteine’s role in mitochondrial ATP synthesis might be associated to its non-oxidative metabolism resulting in H_2_S (via CBS, CSE or MpST) and/or CysSSH (via CARS2) release. H_2_S is the only inorganic compound presenting a bioenergetic role in mammalian cells’ mitochondria (Goubern et al., 2007), that was already reported to contribute to mitochondrial ATP production through the activity of the enzymes involved in cysteine metabolism: MpST in conjunction with CAT (Módis et al., 2013a,b; Abdollahi Govar et al., 2020; Augsburger et al., 2020), CSE (Fu et al., 2012), and CBS (Bhattacharyya et al., 2013; Szabo et al., 2013). At low concentrations (nM), H_2_S is known to stimulate mitochondrial bioenergetics by way of different mechanisms: through donation of electron equivalents to the quinol pool via sulfide:quinone oxidoreductase (SQR); by the glycolytic enzyme glyceraldehyde 3-phosphate dehydrogenase activation, and by persulfidation of ATP synthase (reviewed in Giuffrè and Vicente, 2018). In addition, Li and Yang (2015) reported a role of CSE/H_2_S system in enhancing mtDNA replication and cellular bioenergetics both in smooth muscle cells and mouse aorta tissues. In fact, this can be an explanation for the increased number of mitochondria in ES2 cells under hypoxia, as the mitochondrial marker/endogenous control TOMM20 is overexpressed. More recently, Chakraborty et al. (2018) reported a new role of CBS in the regulation of mitochondria morphogenesis, promoting tumor progression in ovarian cancer. Specifically under hypoxia conditions, H_2_S was reported to decrease reactive oxygen species (ROS), mediated by CBS mitochondrial accumulation (Teng et al., 2013) and induce ATP production, mediated by CSE translocation into the mitochondria (Fu et al., 2012). A different adaptive strategy to hypoxia has been reported by Malagrinò et al. (2019) for the colorectal cancer cell line SW480, which exhibited a lower mitochondrial mass in response to hypoxia, although the mitochondria were enriched in H_2_S-disposal capacity via increased mitochondrial SQR expression. This increased mitochondrial SQR renders hypoxic SW480 cells more equipped to inject H_2_S-derived electron equivalents into the mitochondrial electron transfer chain and sustain mitochondrial bioenergetics.

In our study, short (2–16 h) exposure to CBS and CSE inhibitors did not afford significant differences in ATP levels in both cell lines, with the exception of OVCAR3 cells at 2 h under hypoxia with cysteine supplementation in which the inhibitors led to decreased ATP levels (Supplementary Figure 3A). Hence, OVCAR3 cells, under hypoxia, may channel the extra cysteine to degradation, thereby producing H_2_S and/or CysSSH, and enhancing ATP production. Despite the absent effects at shorter times, the observed increased ATP levels at prolonged (48 h) exposure to CBS and CSE inhibitors indicates that the inhibition of both enzymes allows for compensatory or alternative mechanisms of energy production, probably including CAT/MpST and/or CARS2 activity. Given the fact that both CBS and CSE have been reported to partially relocate to mitochondria in hypoxia (Fu et al., 2012; Teng et al., 2013), available cysteine may be converted in the mitochondria to H_2_S by CBS, CSE and MpST, or to CysSSH by CARS2. Additionally, cystine can be converted to CysSSH and pyruvate by CBS and CSE. Therefore, whereas excess cysteine may trigger over-production of H_2_S and/or CysSSH via CBS and CSE to the point they inhibit the mitochondrial electron transfer chain and thereby impair ATP production, in the presence of their inhibitors AOAA and PAG, this inhibitory effect is likely released and ‘safer’ cysteine-degradation mechanisms can be deployed, linked to stimulation of ATP production.

Under hypoxia we did not observe differences in MpST protein levels in both cell lines (Supplementary Figure 3B), although we observed a mitochondrial MpST enrichment compared to the cytosolic content (Supplementary Figure 3C). Given that cysteine is a poor co-substrate for MpST as sulfane sulfur acceptor to generate CysSSH with respect to other possible co-substrates such as thioredoxin, the excess cysteine load imposed on hypoxic cells may push the CAT/MpST system into higher – yet controlled – CysSSH/H_2_S production. Interestingly, enzymatic studies performed in *E. coli* showed that a bacterial MpST homolog abrogates oxidative stress via L-cysteine (Mironov et al., 2017). Another hypothesis is that diversion of cysteine for CARS2-mediated generation of CysSSH affords a protective role from cysteine oxidation in oxidative stress triggered by hypoxic conditions, as recently shown by Zivanovic et al. (2019) regarding the cysteine persulfidation prevention of protein cysteine oxidative damage.

**FIGURE 7:**
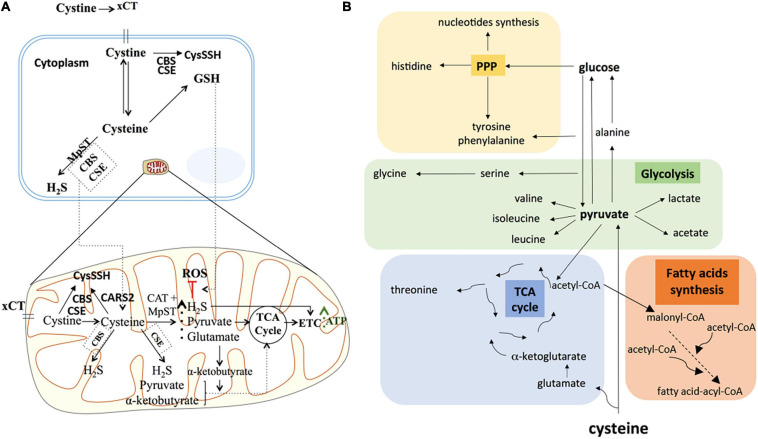
Cysteine possible direct and indirect roles in ATP synthesis and in carbon metabolism reprogramming under hypoxia in ovarian cancer cells. **(A)** Under hypoxia, cysteine degradation could contribute directly to ATP production via not only H_2_S generation, but also via pyruvate and α-ketobutyrate that could further supply the TCA cycle, leading to increased ATP synthesis. Cysteine could also present an indirect role in ATP synthesis mediated by increasing GSH content under hypoxia, hence counteracting oxidative stress and thereby increasing cellular metabolism. Cystine can also be converted into cysteine persulfide (CysSSH) by CSE and CBS in the cytoplasm or by CSE, CBS and CARS2 in the mitochondria. **(B)** The axis cysteine-pyruvate-glucose is central in whole metabolic network of carbon. Cysteine degradation can originate pyruvate and glutamate. Pyruvate besides being a major supplier of tricarboxylic acids (TCA) cycle, it can be converted into valine, isoleucine, leucine, alanine, lactate, and acetate. Alanine can be a source of glucose through gluconeogenesis. Glycolysis produce intermediates to supply the TCA cycle (a hub for many precursors of organic compounds, such as threonine) and the pentose phosphate pathway (PPP). PPP intermediates can be converted into amino acids such as histidine and can be conjugated with glycolysis intermediates, originating tyrosine, phenylalanine and serine, which can be converted into glycine. PPP is also crucial in the nucleotide’s synthesis. Acetyl-CoA is a central organic compound in metabolism, when cysteine-derived it proves that cysteine is a valuable supplier of TCA cycle and amino acids and fatty acids syntheses. The increased concentration of these compounds under hypoxia, suggests that cysteine is pushing the metabolic flow in order to supply the main carbon metabolic pathways. The direct incorporation of cysteine into these compounds is possible, however, further studies are needed to clarify this. Based on information from www.bioinfo.org.cn.

Considering the relevance of cysteine in ATP production, our results showed that cysteine metabolism may operate in a more intricate manner than simply increased H_2_S production and concurrent ATP production. Although some studies showed that NaHS, an exogenous H_2_S source, was sufficient to increase mitochondrial ATP production under hypoxia (Fu et al., 2012), our results showed that H_2_S *per se* was not sufficient to counteract the impaired ATP production driven by sulfasalazine (xCT inhibitor), under hypoxia in both ES2 and OVCAR3 cells. Therefore, our experiments support that cysteine metabolism, dependent on xCT transporter, provides alternative sources for energy production in ovarian cancer cells (Figure 7A).

The synthesis of GSH is another way of cysteine contributing for ATP production. The incorporation of extra cysteine in GSH allows cells to escape from oxidative stress and enables increased cell viability and proliferation, therefore leading to increased ATP synthesis. In fact, our previous data have supported a role of a higher thiols turnover in hypoxia adaptation, especially in ES2 cells (Nunes et al., 2018b). Interestingly, H_2_S was also reported to increase the production of GSH by inducing the expression of cystine/cysteine transporters and by redistributing GSH to mitochondria in mouse neuronal cell models (Kimura et al., 2010).

Despite the fact that in addition to H_2_S and CysSSH production, cysteine and cystine catabolism generate pyruvate as well (Wang, 2012; Bonifácio et al., 2020), cysteine is not commonly considered as a carbon source. Herein, we showed that cysteine gives rise to lactate by being firstly converted into pyruvate and alanine, indicating that cysteine could also be used in gluconeogenesis (Figure 7B). Most importantly, cysteine also originates acetyl-CoA, which is a central metabolite, supplying the TCA cycle, fatty acids synthesis and amino acids synthesis. Our study reinforces that cysteine can account for biosynthesis and bioenergetics not only as a sulfur source but also as a carbon donor.

Importantly, Beaufort et al. (2014) characterized 39 ovarian cancer cell lines in order to correlate the cellular and molecular features with their tumorigenic phenotype. In that study, ES2, but not OVCAR3, was included in the most aggressive subset of ovarian cancer cells (Beaufort et al., 2014). In here, our data supported that cysteine orchestration in metabolic remodeling and plasticity can be a crucial phenomenon for more aggressive cancer cells phenotype. Strikingly, ^1^H-NMR results showed that the metabolic impact of cysteine under hypoxia was much more pronounced in ES2 cells than in OVCAR3 cells, translated by the remarkable number of metabolic pathways that were significantly altered. Therefore, taken together, our results reinforced the role of cysteine as a valuable carbon source, from which cancer cells take advantage on the course of the metabolic rewiring they undergo under hypoxia. In particular, cysteine is a central carbon source for ES2 cells, supporting their redox capacity by supplying GSH synthesis and allowing the maintenance of pivotal biosynthetic and bioenergetic pathways dependent on acetyl-CoA. Furthermore, results suggested that under hypoxia, cysteine allowed to increase the rate of some metabolic pathways in ES2 cells, as increased intracellular levels of several amino acids and other compounds were observed. Regarding OVCAR3 cells, results supported that cysteine impacts differently the cellular needs of amino acids under normoxia and hypoxia. Moreover, when comparing to OVCAR3, results suggest that ES2 cells are better adapted to hypoxia and to the use of cysteine to overcome the hypoxic stress, as seen by the higher number of metabolic pathways that were significantly altered by cysteine under hypoxia.

Interestingly, while cysteine was able to rescue the impaired ATP synthesis triggered by xCT inhibition, it was not able to increase ATP synthesis upon β-oxidation and glycolysis inhibition (Supplementary Figure 3D), indicating that cysteine is not enough to replace the contribution of these pathways for ATP production.

Together, the results support that ES2 and OVCAR3 cells use cysteine differently in order to cope with hypoxia, where cysteine especially impacts hypoxic ES2 cells metabolic features, enhancing the metabolic reprogramming. In Figure 7, we present the possible direct and indirect pathways in which cysteine can be a metabolic coin, promoting ATP production in hypoxic ovarian cancer cells. The profound metabolic impact that cysteine showed under hypoxia in ES2 cells suggests a strong remodeling of the carbon metabolism.

This work lights again that disturbing cysteine metabolic network can be a promising tool not only in ovarian cancer but also in all cancer models that rely their survival on cysteine bioavailability and metabolic versatility.

## Data Availability Statement

The original contributions presented in the study are included in the article/Supplementary Material, further inquiries can be directed to the corresponding author.

## Author Contributions

SN planned and performed most experiments. CR, IS, and FS performed experimental assays. JV coordinated H2S experiments. SP coordinated the pharmacological assays. AF participated in the pathophysiological contextualization of the study. LG coordinated NMR spectroscopy analyses. JS coordinated the whole research project and ensured the funding. CM performed the experiments and contributed for the revised version of the manuscript, which ended up being accepted for publication. All the authors read, discussed, and approved the final version of the manuscript.

## Conflict of Interest

The authors declare that the research was conducted in the absence of any commercial or financial relationships that could be construed as a potential conflict of interest.

## Publisher’s Note

All claims expressed in this article are solely those of the authors and do not necessarily represent those of their affiliated organizations, or those of the publisher, the editors and the reviewers. Any product that may be evaluated in this article, or claim that may be made by its manufacturer, is not guaranteed or endorsed by the publisher.

## Footnotes

1
http://www.hmdb.ca/

2
https://www.metaboanalyst.ca/faces/home.xhtml
