# Supplementary material for: Cysteine Boosts Fitness Under Hypoxia-Mimicked Conditions in Ovarian Cancer by Metabolic Reprogramming
Source: Front Cell Dev Biol. 2021 Aug 11;9:722412. doi: 10.3389/fcell.2021.722412 (PMC8386479; doi:10.3389/fcell.2021.722412)
Supplement: Supplementary file 1 [file Data_Sheet_1.PDF]

Supplement figure 1. Expression of xCT in ES2 and OVCAR3 cells.

A. Western blot analysis with isolated mitochondria for xCT and TOMM20 in ES2 and OVCAR3 cells.

B. Basal relative xCT mRNA expression for ES2 and OVCAR3 cells. Briefly, ES2 and OVCAR3 cells were cultured in 6-well plates ($5x10^5$ cells/well) and cultured under basal conditions (normoxia without cysteine supplementation with 1% of FBS). Cell were collected and RNA was extracted with RNeasy Mini Extraction kit (74104, Qiagen), according to the manufacturer's protocol. cDNA was synthesized from 1µg RNA and reversely transcribed by SuperScript II Reverse Transcriptase (18064-22, Invitrogen), according to the manufacturer's protocol. Quantitative Real-Time PCR was performed using LightCycler 480 SYBR Green I master (04707516001, Roche), according to manufacturer's protocol. Primers for xCT (For: 5' GGTCCTGTCACTATTTGGAGC 3'; Rev: 5' GAGGAGTTCCACCCAGACTC 3') were used. Real-time PCR was carried out in LightCycler 480 instrument (Roche). Results are shown as mean ± SD. *$p<0.05$, **$p<0.01$, ***$p<0.001$ (Independent-samples T test).

Supplement figure 2. ^{1}H-NMR analysis of ES2 and OVCAR3 cells: typical spectra, the effect of cobalt chloride ($CoCl_2$) on histidine levels, and the effect of hypoxia in glucose, lactate, pyruvate, fumarate and glutamine levels.

A. Typical ^{1}H-NMR spectra of the growth media (upper panel) and aqueous phase (bellow panel) of ES2 cells under hypoxia with cysteine. Metabolites: 1- formate, 2- inosine, 3- hypoxanthine, 4- UDP-N-acetyglucosamine, 5- phenyalanine, 6- tyrosine, 7- glucose, 8- lactate, 9- creatine, 10- glutamate, 11-glycine, 12- O-phosphocholine, 13- choline, 14- succinate, 15- acetate, 16- alanine, 17- valine, 18- isoleucine, 19- leucine, 20- nicotinurate, 21- histidine, 22- tryptophan, 23- fumarate, 24- threonine, 25- pyroglutamate, 26- methionine, 27- glutamine, 28- isopropanol, 29- isobutyrate, 30- 2-hydroxybutyrate, 31- methanol. B. Typical ^{1}H-NMR spectra of the growth media (upper panel) and aqueous phase (bellow panel) of OVCAR3 cells under normoxia with cysteine supplementation. Metabolites: 1- formate, 2- inosine, 3- hypoxanthine, 4- UDP-N-acetyglucosamine, 5- phenyalanine, 6- tyrosine, 7- glucose, 8- lactate, 9- creatine, 10- glutamate, 11-glycine, 12- O-phosphocholine, 13- choline, 14- succinate, 15- acetate, 16- alanine, 17- valine, 18- isoleucine, 19- leucine, 20- nicotinurate, 21- histidine, 22- tryptophan, 23- fumarate, 24- threonine, 25- pyroglutamate, 26- methionine, 27- glutamine, 28- isopropanol, 29- isobutyrate, 30- 2-hydroxybutyrate, 31- methanol. C. Histidine levels in control media (without cells) and after 48 h of experimental conditions for ES2 cells. (left panel) and OVCAR3 cells (right panel). D. Glucose, lactate, pyruvate, fumarate and glutamine levels for 48 h of experimental conditions for ES2 (upper panel) and OVCAR3 (below panel) cells. N – Normoxia; NC – Normoxia with cysteine; H – hypoxia and HC – hypoxia with cysteine. In C. and D. results are shown as mean ± SD. *$p<0.05$, **$p<0.01$, ***$p<0.001$ (Independent-samples T test).

Supplement figure 3.

A. Effect of CBS and CSE inhibition in ATP synthesis in ovarian cancer cells. ATP levels in control conditions and in the presence of 1 mM AOOA and 3 mM PAG for 2 h of experimental conditions for ES2 and OVCAR3 cells. The asterisks (*) represent the statistical significance compared to the respective control. *$p<0.05$, **$p<0.01$, ***$p<0.001$ (One-way ANOVA with post hoc Tukey tests). N – normoxia; NC – normoxia with cysteine, H – hypoxia, HC – hypoxia with cysteine. Results are shown as mean ± SD.

B. MpST protein levels in ES2 and OVCAR3 cells. Quantification of anti-MpST western blotting for ES2 and OVCAR3 cells. Data were normalised to control (normoxia). NC – normoxia with cysteine, H – hypoxia, HC – hypoxia with cysteine.

C. Cytosolic and mitochondrial MpST protein levels in ES2 and OVCAR3 cells. Western blotting for cytosolic MpST (left panel) and mitochondrial MpST (right panel) in ES2 and OVCAR3 cells. For western blotting for cytosolic MpST, briefly, cells (2.5×10^6) were cultured in

25-cm2 tissue culture flasks in control conditions and exposed either to 0.402 mM L-cysteine and/or 0.100mM cobalt chloride for 16 h. Cells were collected with trypsin and western blot analysis was performed. Anti-MpST (1:250; HPA001240 from sigma) and anti-β-actin (1:5000; A5441 from Sigma Aldrich) antibodies were used. Secondary antibodies (1:5000; anti-rabbit, 31460, from Thermo Scientific or anti-mouse 31430 from Thermo Scientific) were used. For the western blotting presented, 100 μg of total protein was used. For western bloting with isolated mitochondria, the methods are described previously in the materials and methods section.

D. **Cysteine is not able to rescue ATP production upon β-oxidation and glycolysis inhibition.** ATP levels for 48 h of experimental conditions for ES2 (left panel) and OVCAR3 (right panel) cells under hypoxia with and without cysteine and in the presence of the β-oxidation inhibitor etomixir and glycolysis inhibitor, bromopyruvic acid. Data were normalised to the respective control condition (the same environmental condition H/HC without etomoxir or bromopyruvic acid). H – hypoxia, HC – hypoxia with cysteine. Results are shown as mean ± SD. *p<0.05, **p<0.01, ***p<0.001 (Independent-samples T test).
