# Supplementary figures and images for: Cysteine Boosts Fitness Under Hypoxia-Mimicked Conditions in Ovarian Cancer by Metabolic Reprogramming
Source: Front Cell Dev Biol. 2021 Aug 11;9:722412. doi: 10.3389/fcell.2021.722412 (PMC8386479; doi:10.3389/fcell.2021.722412)

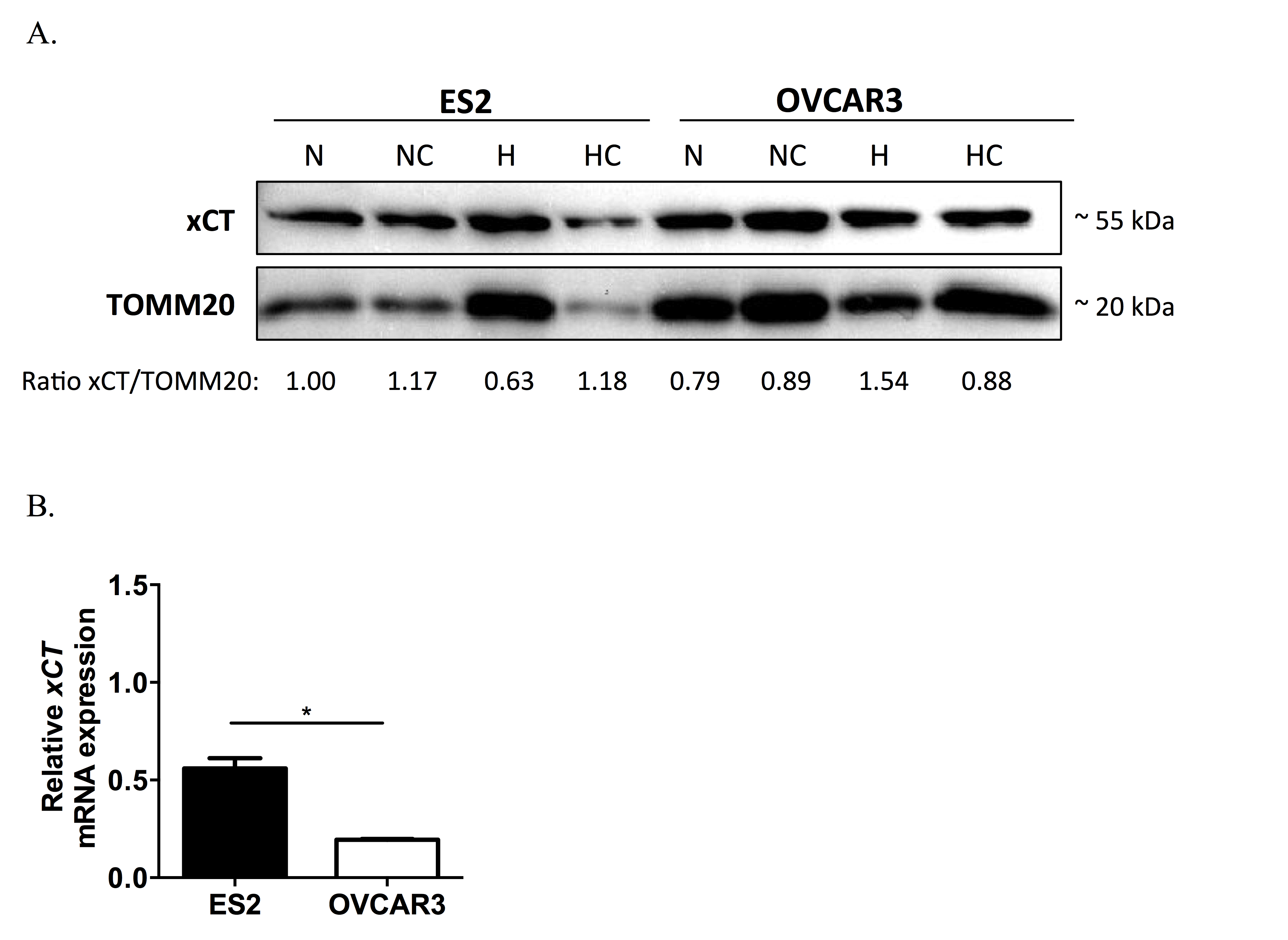

Supplement: Supplementary file 2 [file Image_1.TIFF]

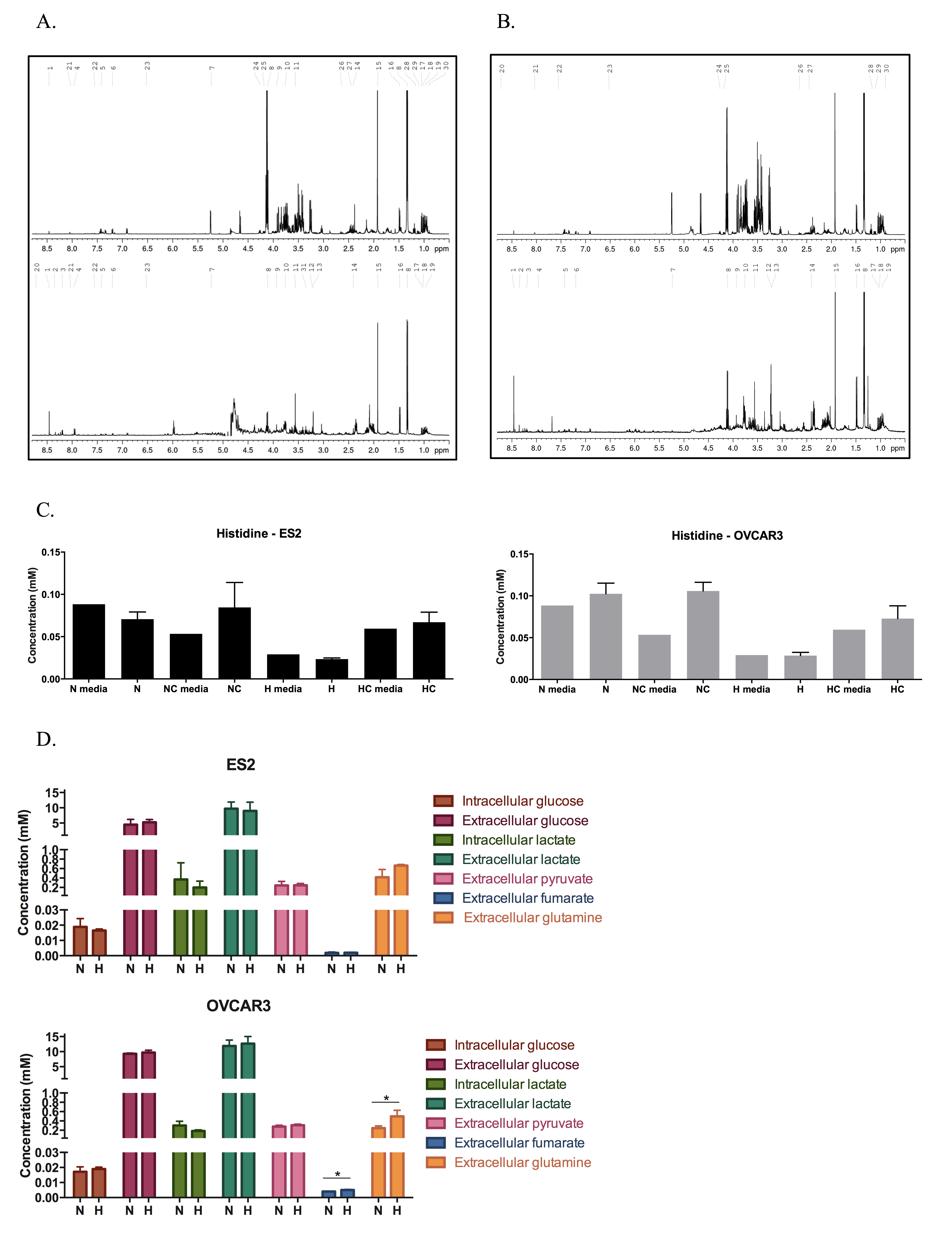

Supplement: Supplementary file 3 [file Image_2.TIFF]

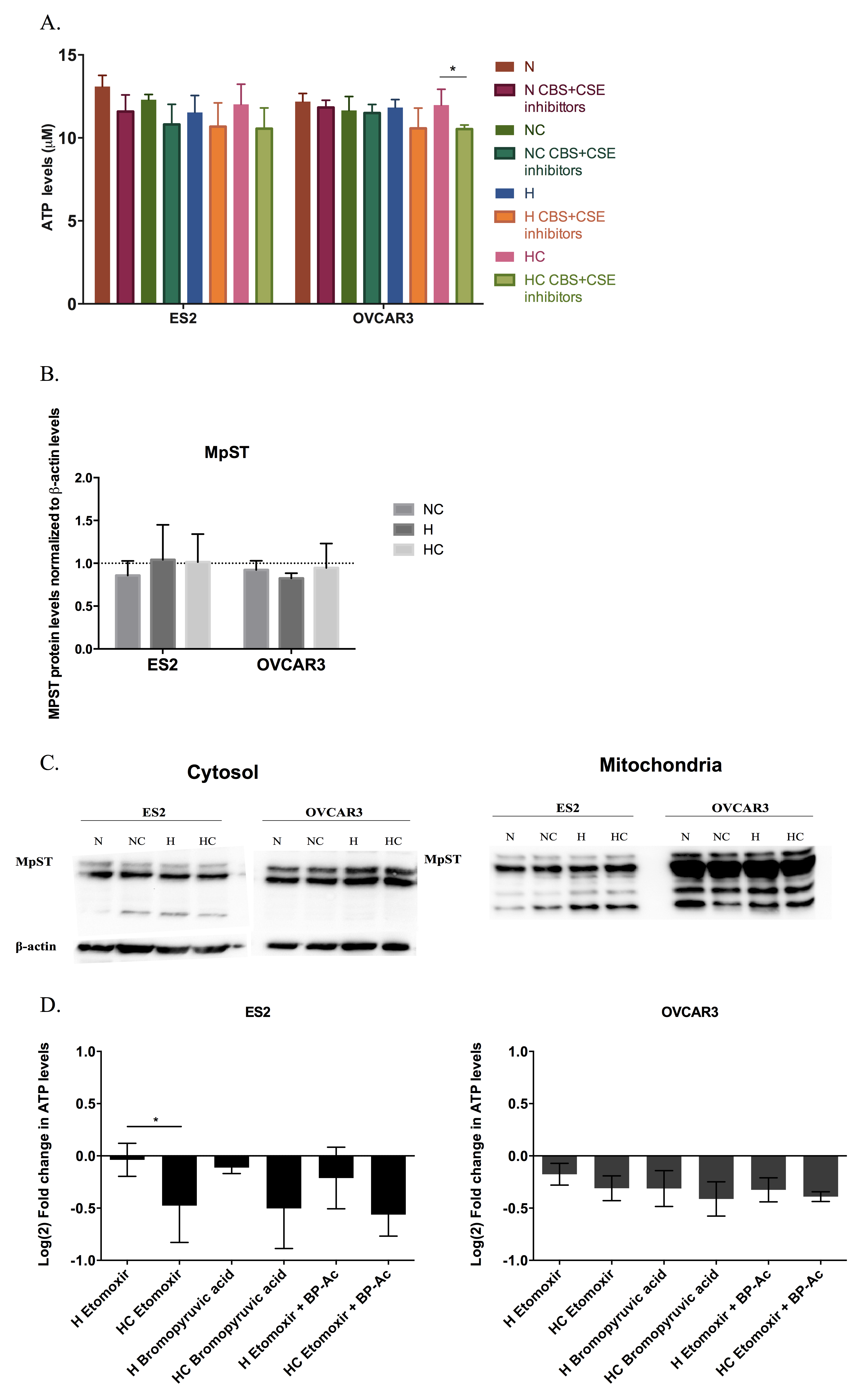

Supplement: Supplementary file 4 [file Image_3.TIFF]
